# Comparison of next-generation sequencing (NGS) and next-generation flow (NGF) for minimal residual disease (MRD) assessment in multiple myeloma

**DOI:** 10.1038/s41408-020-00377-0

**Published:** 2020-10-30

**Authors:** Alejandro Medina, Noemi Puig, Juan Flores-Montero, Cristina Jimenez, M.-Eugenia Sarasquete, María Garcia-Alvarez, Isabel Prieto-Conde, Carmen Chillon, Miguel Alcoceba, Norma C. Gutierrez, Albert Oriol, Laura Rosinol, Joan Bladè, Mercedes Gironella, Miguel T. Hernandez, Veronica Gonzalez-Calle, Maria-Teresa Cedena, Bruno Paiva, Jesus F. San-Miguel, Juan-Jose Lahuerta, Maria-Victoria Mateos, Joaquin Martinez-Lopez, Alberto Orfao, Marcos Gonzalez, Ramon Garcia-Sanz

**Affiliations:** 1 Departamento de Hematología, Hospital Universitario de Salamanca (HUSA/IBSAL), CIBERONC, CIC-IBMCC (USAL-CSIC), Salamanca, Spain; 2 Centro de Investigación del Cáncer-IBMCC (USAL-CSIC), CIBERONC, Salamanca, Spain; 3 Hospital Germans Trias i Pujol, Institut Català d’Oncología (ICO), Institut Josep Carreras, Badalona, Spain; 4 Hospital Clínic i Provincial, Institut de Investicacions Biomediques August Pi i Sunyer (IDIBAPS), Barcelona, Spain; 5 Hospital Vall d’Hebrón, Barcelona, Spain; 6 Hospital Universitario de Canarias, Santa Cruz de Tenerife, Spain; 7 Hospital 12 de Octubre, i + 12, CNIO, Universidad Complutense, Madrid, Spain; 8 Clínica Universidad de Navarra (CUN), Centro de Investigación Médica Aplicada, IDISNA, CIBERONC, Pamplona, Spain

**Keywords:** Myeloma, Risk factors

## Abstract

Detecting persistent minimal residual disease (MRD) allows the identification of patients with an increased risk of relapse and death. In this study, we have evaluated MRD 3 months after transplantation in 106 myeloma patients using a commercial next-generation sequencing (NGS) strategy (LymphoTrack®), and compared the results with next-generation flow (NGF, EuroFlow). The use of different marrow pulls and the need of concentrating samples for NGS biased the applicability for MRD evaluation and favored NGF. Despite that, correlation between NGS and NGF was high (*R*^2^ = 0.905). The 3-year progression-free survival (PFS) rates by NGS and NGF were longer for undetectable vs. positive patients (NGS: 88.7% vs. 56.6%; NGF: 91.4% vs. 50%; *p* < 0.001 for both comparisons), which resulted in a 3-year overall survival (OS) advantage (NGS: 96.2% vs. 77.3%; NGF: 96.6% vs. 74.9%, *p* < 0.01 for both comparisons). In the Cox regression model, NGS and NGF negativity had similar results but favoring the latter in PFS (HR: 0.20, 95% CI: 0.09–0.45, *p* < 0.001) and OS (HR: 0.21, 95% CI: 0.06–0.75, *p* = 0.02). All these results reinforce the role of MRD detection by different strategies in patient prognosis and highlight the use of MRD as an endpoint for multiple myeloma treatment.

## Introduction

Multiple myeloma (MM) is a B-cell malignancy where plasma cells arise from a single clone accumulated in the bone marrow^1^, and usually produce monoclonal immunoglobulins (M-protein or monoclonal component). New agents such as proteasome inhibitors and immunomodulatory drugs^2,3^, together with autologous stem cell transplantation (ASCT)^4^ have increased the proportion of patients achieving complete response (CR) in this disease, which has been translated into better progression-free survival (PFS) and overall survival (OS) rates during the last 10 years^5–7^. This improvement led to the definition of new response categories (i.e. stringent complete response, sCR)^8^, but even myeloma patients with such good responses still relapse due to the presence of residual tumor cells in the bone marrow (i.e. minimal residual disease, MRD)^9–11^ that remain undetectable by conventional serological and morphological methods. New methods are capable of detecting MRD with a higher prediction ability, since negative patients had longer survival rates compared to positive patients^12–14^, even when only patients in CR are considered^15^. Accordingly, the International Myeloma Working Group (IMWG) developed new criteria to define MRD negative responses, characterized by the absence of clonal malignant plasma cells in patients with suspected CR, assessed with a sensitivity of at least 10^−5^—one malignant cell per hundred thousand normal cells^16^; therefore, only very sensitive and reliable methods can be applied to detect MRD.

Nowadays, MRD in MM can be studied using two different approaches: immunophenotypic (multiparametric flow cytometry, MFC)^17^ and molecular techniques (next-generation sequencing —NGS—, digital PCR)^18^. Imaging tools (PET-CT, MRI) can add some extra information in terms of prognostic evaluation^19^, however, standardized procedures to perform MRD assessment in myeloma have only been developed for MFC^20^ and NGS^21^. Despite the added value for MRD assessment, next-generation strategies are currently used only in a few centers and usually limited to the context of clinical trials.

MFC allows the identification and quantification of abnormal plasma cells based on an aberrant protein-marker expression profile displayed by myeloma cells. Recently, the EuroFlow consortium has developed a two-tube, eight-color flow assay that allows the simultaneous analysis of up to 10 million cells (next-generation flow, NGF)^22^; NGF has been already approved by the IMWG as a reference method to detect and define immunophenotypic CRs in MM^16^. NGF reaches a sensitivity of 2 × 10^−6^, overcoming previous flow protocols (10^−4^–10^−5^), but it is highly dependent on the precise identification of the pathologic immunophenotype, requiring a high level of expertise; in addition, the quality of the sample must be high and should be promptly processed^23^.

Molecular methods use the clonal immunoglobulin gene rearrangement as target for the detection of MRD levels. Quantitative PCR uses allele-specific oligonucleotides targeting the junction region of the immunoglobulin genes; it has been extensively used as the molecular gold-standard approach to detect MRD levels in lymphoid malignancies^24,25^. Nonetheless, it is a labor-intensive technique, requiring the construction of a standard curve for every single patient, and its applicability accounts only for 40–75% of myeloma patients^24,25^. By contrast, NGS strategies have been rapidly introduced in the clinical setting, especially for myeloma. However, the only strategy approved by the IMWG^16^ and cleared by the FDA —Adaptive’s clonoSEQ®— is not commercially available, and its cost makes it unaffordable for most centers.

We aimed at validating the potential applicability and usefulness of a new NGS methodology for the diagnosis and MRD detection in MM patients. To that end, we have evaluated a commercial NGS panel, LymphoTrack®, in a cohort of 106 myeloma patients, and compared the results with NGF.

## Materials and methods

### Patient and sample selection

Patient selection was based on the following criteria: (i) newly diagnosed MM, transplant-candidate patients included in the Spanish GEM2012 clinical trial^7^, whose clonotypic rearrangements were previously identified, (ii) NGF evaluation performed at diagnosis and follow-up per protocol, and (iii) enough amount of gDNA available for MRD studies. Bone marrow samples from eligible MM patients were collected at day 100 following transplantation: two independent pulls were obtained and subsequently processed to perform flow and molecular studies.

Patients with t(4;14), t(14;16), or del17p by FISH were grouped together in a high-risk cytogenetic subgroup according to the IMWG criteria^26^. The revised international staging system (R-ISS)^27^ was also applied to stratify patients according to the ISS stage, serum LDH, and cytogenetics. The study was conducted in accordance with Declaration of Helsinki principles, was approved by the Committee of the University Hospital of Salamanca, and written informed consent was required and obtained from each patient prior to their inclusion.

### DNA extraction and quantification

gDNA was isolated from bone marrow aspirates of newly diagnosed MM patients using the automated DNA Purification kit Maxwell® (Promega, Madison, WI, USA). gDNA quality was first assessed using NanoDrop2000 (ThermoFisher, Waltham, MA, USA). Samples were quantified using Qubit 2.0 and the dsDNA BR assay (ThermoFisher, Waltham, MA, USA).

To improve the quality of follow-up samples, those with insufficient DNA concentration for MRD purposes (i.e. those with <100 ng/µl) were ethanol-precipitated. To do this, 1/10 volume sodium acetate, as well as twice the sample volume of 100% ethanol (stored at −20 °C) were added to the samples and incubated overnight at −20 °C. Afterwards, samples were centrifuged at 17,900 × *g* and 4 °C for 10 min. The nucleic acid pellet was then washed with 500 µl ethanol (70%) and centrifuged again at 17,900 × *g* and 4 °C for 5 min. Finally, the pellet was dried and rehydrated in ≈12 µl of water, and quantified using the Qubit dsDNA BR assay.

### Gene amplification and sequencing

The LymphoTrack® IGH panel (Invivoscribe Technologies, San Diego, CA, USA) was used for the analysis of MRD samples to detect previously characterized clonotypic rearrangements of the immunoglobulin heavy chain loci (IGH). Clonal rearrangements were first determined in matched baseline samples by PCR amplification and Sanger sequencing using the BIOMED-2 primers as previously described^28^.

Briefly, this commercial NGS strategy uses primers targeting the immunoglobulin framework regions in order to amplify V(D)J rearrangements. In one-step PCR (Fig. S1) amplicons are generated and one-side indexed, allowing the simultaneous sequencing of up to 24 samples in a single run. Whenever possible, ≥650 ng of DNA were used to reach a sensitivity level of at least 10^−5^ (assuming 6.5 pg of DNA per cell, input for NGS would be at least 100,000 cells). In addition, DNA from one clonal, well-characterized B cell line was added in each reaction as a control spike-in (corresponding to 100 cells), to allow the absolute quantification of tumor plasma cells.

After a purification step with Agentcourt AMPure XP microbeads (Beckman Coulter Inc., Brea, CA, USA) and 70% ethanol, and purity and quantity assessment using the TapeStation 4200 (Agilent, Santa Clara, CA, USA) and KAPA library quantification kit (KAPA Biosystems, Boston, MA, USA) or Qubit 2.0, respectively, amplicon libraries of 12–20 pM were prepared. These libraries were later sequenced in a MiSeq platform (Illumina, San Diego, CA, USA) using v3 reagent kits and 2 × 251 sequencing cycles, aiming at one million reads per sample.

### Sequence analysis and MRD evaluation

Resulting FastQ files were processed using the LymphoTrackAnalysis® software and the LymphoTrack® MRD Data Analysis tool 1.1.0 (Invivoscribe Technologies) to retrieve sequences from virtually every clonal B cell of the samples. This allowed the identification of residual tumor cells, if present, by tracking their clonotypic *IGH* complementarity-determining region 3 (CDR3) that had previously been characterized. The software performs a calculation considering the number of spike-in cells used for each reaction, as well as tumor and spike-in read counts. Sequencing results were considered invalid when <20,000 total reads were obtained. A sample was considered positive for MRD assessment when at least two identical clonotypic reads were detected; conversely, a sample was considered negative (undetectable) for MRD when criteria for positivity were not met. Vidjil (University of Lille) and Arrest/Interrogate (EuroNGS) tools were also used to analyze FastQ files from MRD sequencing.

In order to make comparisons, patients were eventually classified into different groups according to the MRD status (MRD-positive and MRD-negative), the MRD level (MRD-negative, MRD-positive below 10^−5^, MRD-positive between 10^−5^ and 10^−4^, and MRD-positive higher than 10^−4^), the conventional response (VGPR/PR and sCR/CR), and the cytogenetic risk (high- and standard-risk).

### MFC studies

Follow-up samples were processed within 24 h after collection. Analysis was carried out using the recently developed NGF methodology (Table S1), following the EuroFlow guidelines, as described elsewere^20,22^. Events from two eight-color tubes (per sample) were merged using the merge function of the INFINICYT™ v2.0 software (Cytognos S.L. Salamanca, Spain). A sample was considered positive when at least 20 aberrant plasma cells were detected. Hemodilution of bone marrow samples for NGF evaluation was assessed through the identification of a significant decrease in non-plasma cell populations: mast cells (CD117^hi^), erythroblasts (CD45^−^/sideward-scatter^lo^) and B-cell precursors (CD19^+^/CD38^hi^/CD45^lo^). The complete analysis of NGF performance for the GEM2012 trial has already been published^28^.

### Statistical analysis

Patients’ characteristics were analyzed with the SPSS 23.0 software (IBM, Armonk, NY, USA) using Fisher’s exact test for discrete variables and the Mann–Whitney test for continuous variables. Bland–Altman plots were used to test the potential agreement between methods. The Kaplan–Meier method and the log-rank test were used to plot and compare PFS and OS curves. Cox regression was used to perform univariate and multivariate analyses. By Landmark analysis, only those patients who had not progressed or died on day 100 post ASCT were evaluated. PFS was defined as the time from MRD assessment to the last follow-up visit, disease progression or death by any cause. OS was defined as the time from MRD assessment to the last follow-up visit or patient’s decease by any cause. All reported *p* values were obtained by a two-sided exact method, at the conventional 5% significance level (*p* < 0.05).

## Results

### Patient characteristics

One hundred and six (106) patients met the inclusion criteria. Clinical variables of our cohort are described in Table S2. Median age at diagnosis was 59 years. Male to female proportion was 58/42. Patients with high-risk cytogenetics represented 22.5% of the present series [*n* = 23/102, one of them with t(4;14) plus del17p]. The R-ISS stages I, II, and III represented 25.2%, 59.2%, and 15.5% of patients, respectively. After a median follow-up of 39.5 months from MRD assessment (interquartile range: 33.8–46.2 months), 31 patients had relapsed (29.2%) and 16 had died (15.1%), two of them without progression, due to infections.

At the corresponding MRD evaluation time point, the overall response rate was 96.2%: 32.1% of patients achieved sCR, 23.6% CR, 34.0% very good partial response (VGPR) and 6.6% partial response (PR). Only four patients (3.8%) had stable/progressive disease (SD/PD). Three-year PFS and OS rates were 72.5% and 86.7%, respectively.

### Comparison of NGS and NGF performance

Sample quality and ethanol precipitation were the main variables affecting NGS applicability for MRD analysis. Because of that, the minimum number of cell equivalents to reach a virtual sensitivity of 10^−5^ could only be used in 95 (89.6%) cases (Table 1). According to the inclusion criteria, NGF was successfully used for the evaluation of the entire series, with a significantly higher number of cells required to perform the studies at the aimed sensitivity threshold. The analysis of precursor B cells, erythroblasts, and mast cells did not reveal significant hemodilution of any sample evaluated by flow cytometry.

**Table 1:** Cell input for NGS- and NGF-based MRD studies.

Input cells	NGS	NGF
<10^5^	10.4%^a^	0%
10^5^ − 2 × 10^5^	32.1%	0%
>2 × 10^5^	57·5%	100%
>10^6^	0%	100%
Median cell input [Interquartile range]	210,672 [136,382–264,110]	9,200,276 [5,702,369–10,305,207]
Median sensitivity	10^−5^	2 × 10^−6^
Cell input range	3542–726,264	1,548,175–15,000,000

Each cell shows the frequency of samples that were analyzed at different cell input levels. The number of cell equivalents used for NGS was calculated based on Qubit2.0 quantification, assuming 6.5 pg DNA/cell.

^a^One sample was sequenced with a cell input below 10,000 cells.

MRD-negativity was achieved by 53 (50.0%) and 58 (54.7%) patients by NGS and NGF, respectively. Further stratification of patients according to the MRD level is shown in Table 2.

**Table 2:** MRD stratification for next-generation flow and next-generation sequencing.

MRD level	Current study		GEM2012^7^
	NGS	NGF	NGF
Negative	53 (50.0%)	58 (54.7%)	173 (54.6%)
<10^−5^	16 (15.1%)^a^	9 (8.5%)	18 (5.7%)^a^
≥10^−5^ and <10^−^^4^	17 (16.0%)	21 (19.8%)	65 (20.5%)
≥10^−^^4^	20 (18.9%)	18 (17.0%)	61 (19.2%)
Total	106	106	317

MRD results, obtained using NGS or NGF, were compared for the 106 patients in our cohort. Results obtained in the GEM2012 Spanish trial,^7^ (intent-to-treat population) were used as a reference. Absolute numbers and percentage (in parenthesis) for each MRD category are shown in each cell.

*MRD* minimal residual disease, *NGF* next-generation flow, *NGS* next-generation sequencing.

^a^Fisher’s exact test showed statistical differences in the proportions of each subgroup (two-sided *p* = 0.004).

**Fig. 1:**
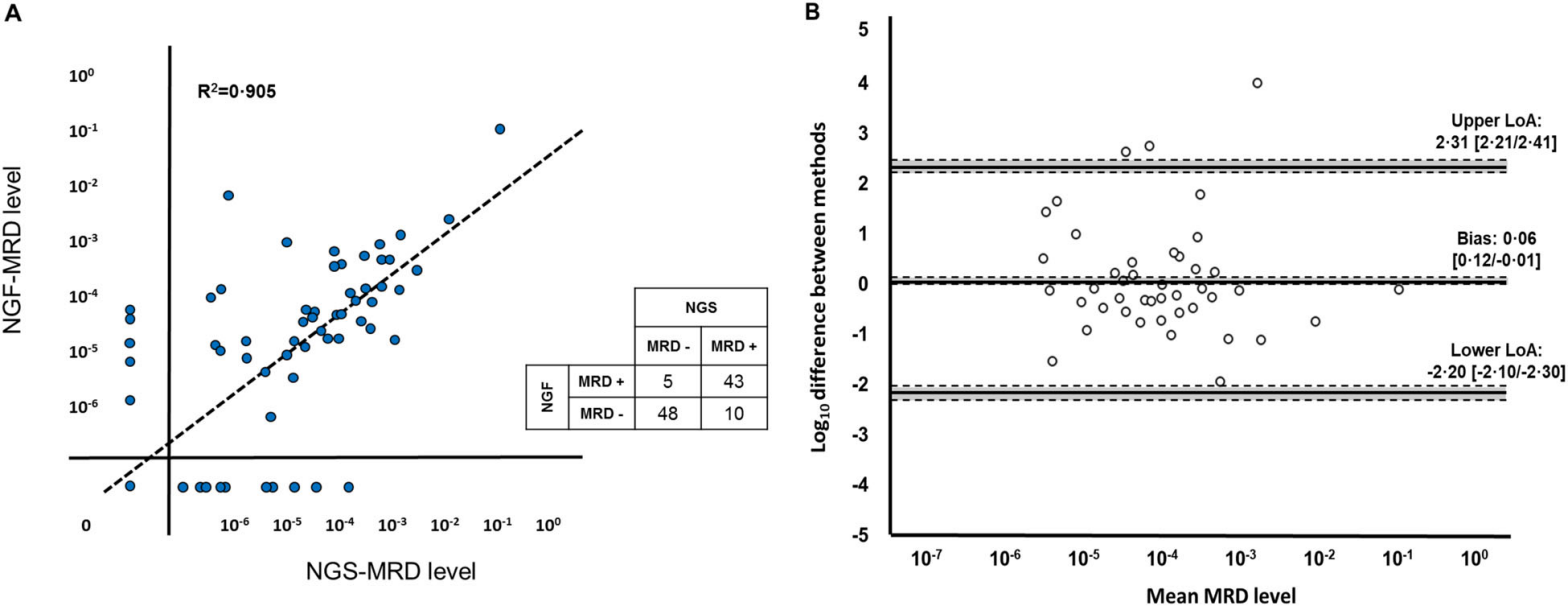
Comparison of MRD results. **A** Linear regression. Ninety-one out of one hundred and six samples (91/106, 85.8%) were concordant between techniques. Only 15/106 cases (14.2%) were discordant. **B** Bland–Altman plot comparing NGS and NGF performance (*n* = 43, only double-positive cases were evaluated). Mean MRD values of methods (shown in the *x*-axis) were calculated. Differences in log_10_ scale for each case (*y*-axis) were calculated as follows: log_10_(higher MRD value/lower MRD value). Then, negative values were assigned to those cases where the MRD level estimated by NGS > NGF, while positive values were assigned to those case where the MRD estimated by NGS < NGF. Normal distribution of the differences was first assessed (Kolmogorov–Smirnov’s *p* > 0.05, *n* = 42 degrees of freedom). The Student’s *T*-test (*t* = 0.33, SD = 1.15; *n* = 42 degrees of freedom) was used to calculate the average of differences (bias), where a positive value indicates a general overestimation made by NGF, and a negative value indicates an overestimation made by NGS. Upper and lower limits of agreement were calculated as the bias ± 1.96 multiplied by the standard deviation of the differences. 95% confidence interval limits for mean and agreement limits are represented as gray shades. Overall, the bias was non-significant (mean: 0.06, *p* > 0.05), which means that the average estimation made by NGF is 10^0.06^ or 1.15 times higher than that made by NGS. Differences between methods were homogenously distributed across the range of MRD levels (*x*-axis), with the limits of agreement approximately set in ±10^2^ and only 3/43 cases (7%) outside the acceptable range.

As it is shown in Fig. 1A, we found a good correlation between methods (*r* = 0.951; *R*^2^ = 0.905), with 15 discordant cases (5 NGF+/NGS−; 10 NGF−/NGS+). It is worth mentioning that the majority of NGS-only MRD-positive cases (7/10) presented values below 10^−5^, while this situation was found in two out of five NGF-only MRD-positive cases. Bland–Altman analysis demonstrated an excellent level of agreement between NGS and NGF (Fig. 1B).

### Characteristics of patients with undetectable MRD who had disease progression

With a median follow-up of 39.5 months, disease progression was confirmed in 31 cases: 23 double-positive patients, three discordant cases (two positive cases by NGF, one positive case by NGS), and five patients with double-negative MRD (by both NGS and NGF).

Table S3 lists the characteristics of the five patients who had undetectable MRD and progressed: all of them achieved CR at the time of MRD assessment, had low/intermediate ISS status and LDH was normal. Of note, two of these patients had extramedullary disease (one already present at diagnosis), while another one relapsed after more than 3 years in sustained MRD negativity, one year following their treatment suspension. The other two patients had conventional disease progressions ~36 months after transplantation.

### Undetectable MRD was clinically significant by NGS and NGF

**Fig. 2:**
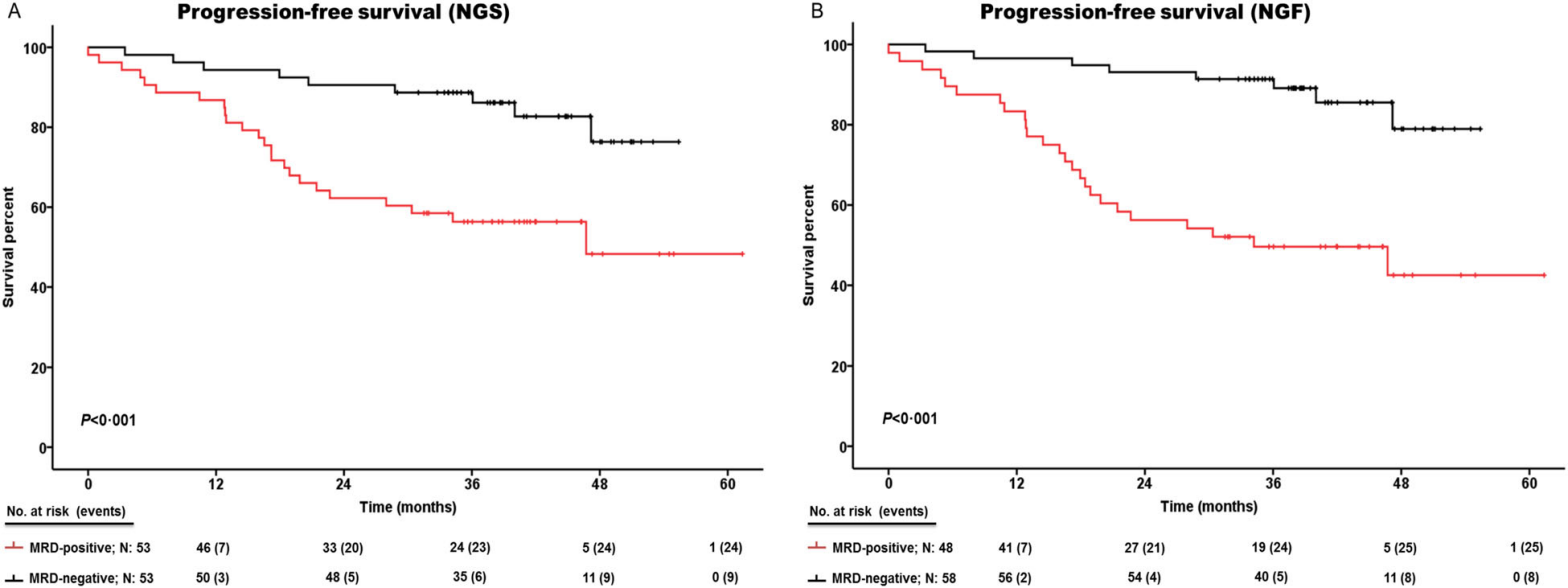
Kaplan–Meier curves comparing progression-free survival of MRD-positive and MRD-negative subsets. **A** Progression-free survival of NGS-based results. **B** Progression-free survival of NGF-based results. Time was calculated from the time of MRD assessment, 3 months after transplantation. Negative patients are represented in black; positive patients are represented in red. Patients at risk are shown at each time point below plots; events are represented between parentheses. Median PFS of positive patients was 46.7 and 34.2 months for NGS and NGF, respectively. Median PFS was not achieved by negative patients. MRD minimal residual disease, NGF next-generation flow, NGS next-generation sequencing.

**Fig. 3:**
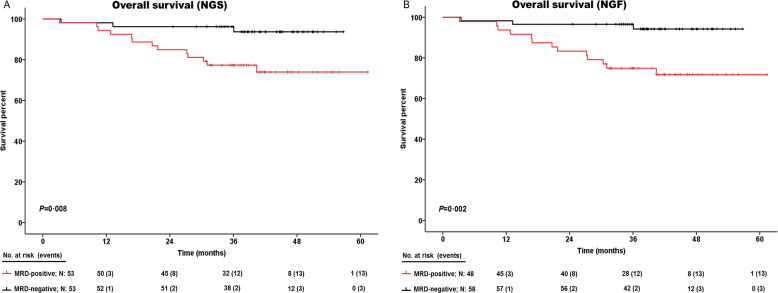
Kaplan–Meier curves comparing the overall survival of MRD-positive and MRD-negative subsets. **A** Overall survival of NGS-based results. **B** Overall survival of NGF-based results. Time was calculated from the time of MRD assessment, 3 months after transplantation. Negative patients are represented in black; positive patients are represented in red. Patients at risk are shown at each time point below plots; events are represented between parentheses. Median overall survival was not achieved by positive or negative patients (3-year OS rates of NGS-positive vs. NGS-negative patients: 77.3% vs. 96.2%; 3-year OS rates of NGF-positive vs. NGF-negative patients: 74.9% vs. 96.6%). MRD minimal residual disease, NGF next-generation flow, NGS next-generation sequencing.

Negative patients showed a significantly better 3-year PFS rate than positive patients (*p* < 0.001), either basing on NGS or NGF results (88.7% vs. 56.6%; 91.4% vs. 50%, Fig. 2A and B, respectively, *p* < 0.001). Three-year OS rates were very high for all subgroups: considering NGS, 3-year OS rates of negative and positive patients were 96.2% and 77.3%, respectively (Fig. 3A; *p* < 0.01). Considering NGF, 3-year OS rates of negative and positive patients were 96.6% and 74.9%, respectively (Fig. 3B; *p* < 0.01). There were no statistical differences in PFS between double-negative and discordant cases (36-month PFS: 91.7% and 80%, respectively, *p* = 0.55, Fig. S2), while both subgroups had significantly better PFS rates than double-negative cases (median PFS: 34.2 months, *p* < 0.05 for both comparisons).

Moreover, survival rates progressively increased from patients with high-positive MRD to patients with undetectable residual disease (Fig. S3): by NGS, 36-month PFS rates of ≥10^−4^, 10^−5^ − 10^−4^, <10^−5^ and negative groups were 55%, 52.9%, 62.5%, and 88.7%, respectively (*p* < 0.01). By NGF, they were 38.9%, 47.6%, 77.8%, and 91.4%, respectively (*p* < 0.001). OS rates of these subsets followed the same trend.

### MRD overcomes response-to-treatment and cytogenetics as a predictive factor

As in prior reports, we correlated the MRD status with different clinical variables such as heavy and light chains usage, ISS, R-ISS, cytogenetics, and conventional response. We only detected a significant association between achieving CR/sCR and MRD negativity (Fig. S4). Thus, by NGS we observed that 71.2% of patients in CR/sCR were MRD negative, while only 23.3% of patients in VGPR/PR achieved MRD negativity (*p* < 0.0001); when NGF data were used, 81.4% of patients in CR/sCR were MRD-negative versus only 20.9% of patients in VGPR/PR (*p* < 0.0001). No differences were observed in the proportion of each group when both techniques were compared (*p* > 0.05).

A significant relationship between negative MRD and longer PFS was observed irrespective to the response (Fig. S5) when patients achieving at least VGPR were considered (*n* = 95). Remarkably, MRD-negative patients achieving VGPR showed comparable 3-year PFS rates to those achieving CR or sCR; this result was similar either considering NGS (VGPR: 88.9%, CR/sCR: 88·1%; *p* > 0.05) or NGF data (VGPR: 100%, CR/sCR: 89.6%; *p* > 0.05). On the other hand, MRD-positive patients had inferior outcomes, with no differences observed between responses.

Then, we stratified patients (*n* = 102) into four groups considering the MRD status (positive or negative) and cytogenetics (high-risk or standard-risk). MRD-negative patients showed excellent 3-year PFS rates independently of their cytogenetic status (Fig. S6), while the outcome of MRD-positive patients was significantly determined by their cytogenetic profile: 3-year PFS rates were prolonged for standard-risk patients (67.1% by NGS, 61.8% by NGF), while for high-risk patients it was 28.6% by NGS and 15.4% by NGF (median PFS: 14.5 months by NGS and 12.8 months by NGF, *p* < 0.01 compared to the other groups). For OS (Fig. S7), all MRD-negative patients showed comparable 3-year survival rates to those patients showing MRD positivity but no high-risk cytogenetic alteration (OS rates >90% for all categories, *p* > 0.05 for all paired comparisons); conversely, survival rates of high-risk, MRD-positive patients were significantly lower than the other subgroups (median OS: 21.7 months for NGS and NGF, *p* < 0.01 for all paired comparisons). Interestingly, 55% (6/11) of t(4;14) patients achieved MRD negativity, while only 12.5% (1/8) of patients with del17*p* were MRD negative.

### Univariate and multivariate analyses

When the univariate analyses for PFS and OS were performed (Table 3), the MRD status detected by NGS and NGF were amongst the most statistically significant variables associated with the outcome: hazard ratios (HRs) for PFS according to the MRD status after transplantation were 0.20 for NGF (95% CI: 0.09–0.44, *p* < 0.001) and 0.29 for NGS (95% CI: 0.14–0·63, *p* = 0.002); HRs for OS were 0.18 for NGF (95% CI: 0.05–0.62, *p* = 0.007) and 0.21 for NGS (95% CI: 0.06–0.75, *p* = 0.02).

**Table 3:** Univariate analysis for progression-free and overall survival.

Variable (*N*)	Univariate analysis for PFS			Univariate analysis for OS		
	Median survival	HR [95% CI]	*p*	Median survival	HR [95% CI]	*p*
*Sex*						
Male (61)	NA	1.36 [0.68–2.78]	>0.05	NA	1.01 [0.38–2.71]	>0.05
Female (45)	NA			NA		
*Cytogenetic risk*						
High risk (23)	40.02	2.99 [1.45–6.16]	0.003	NA	6.33 [2.24–17.86]	<0.001
Standard risk (79)	NA			NA		
*LDH*						
High (20)	NA	1.73 [0.80–3.72]	>0.05	NA	2.00 [0.69–5.75]	>0.05
Low (86)	NA			NA		
*ISS stage*						
I or II (70)	NA	0.63 [0.31–1.31]	>0.05	NA	0.22 [0.07–0.67]	<0.01
III (33)	NA			NA		
*R-ISS stage*						
I or II (87)	NA	0.34 [0.16–0.74]	0.008	NA	0.12 [0.04–0.44]	<0.001
III (16)	34.20			36.1		
*Conventional response*						
sCR/CR (59)	NA	0.54 [0.27–1.08]	>0.05	NA	0.36 [0.12–1.04]	>0.05
VGPR/PR (43)	NA			NA		
*MRD status (NGF)*						
Negative (58)	NA	0.20 [0.09–0.44]	<0.001	NA	0.18 [0.05–0.62]	0.007
Positive (48)	34.20			NA		
*MRD status (NGS)*						
Negative (53)	NA	0.29 [0.14–0.63]	0.002	NA	0.21 [0.06–0.75]	0.02
Positive (53)	46.72			NA		

Cox’s proportional hazards model was used to test individual variables. The number of patients corresponding to each variable’s category is shown in parenthesis. Hazard ratios were calculated comparing first vs. second subgroup of each variable.

*CI* confidence interval, *CR* complete response, *HR* hazard ratio, *ISS* international staging system, *LDH* lactate dehydrogenase, *MRD* minimal residual disease, *NA* not achieved, *NGF* next-generation flow, *NGS* next-generation sequencing, *PR* partial response, *R-ISS* revised international staging system, *sCR* stringent complete response, *VGPR* very good partial response.

In the multivariate Cox regression models (Table 4), MRD negativity achieved by NGF (HR: 0.20, 95% CI: 0.09–0.45, *p* < 0.001) and the R-ISS I or II (HR: 0.37, 95% CI: 0.17–0.78, *p* = 0.01) were associated with the lowest risk of progression or death with independent value. Similarly, MRD negativity achieved by NGF was significantly associated with a decreased risk of death (HR: 0.21, 95% CI: 0.06–0.75, *p* = 0.02), along with the R-ISS (HR: 0.13, 95% CI: 0.05–0.38, *p* < 0.001). MRD negativity by NGS had similar results but it was removed from the stepwise analysis because of its close association with MRD by NGF. Nonetheless, when the analysis was done using the 10^-5^ cutoff, considering only the 95 patients not affected by preanalytical pitfalls, MRD negativity by NGF (HR: 0.16, 95% CI: 0.07–0.37, *p* < 0.001) and the R-ISS (HR: 0.30, 95% CI: 0.12–0.72, *p* = 0.007) were still selected for PFS, while MRD negativity by NGS (HR: 0.15, 95% CI: 0.04–0.57, *p* = 0.005) and the R-ISS (HR: 0.13, 95% CI: 0.04–0.39, *p* < 0.001) were selected for OS, indicating a similar potential for prediction.

**Table 4:** Multivariate analysis for progression-free and overall survival.

Variable	Multivariate analysis for PFS		Multivariate analysis for OS	
	HR [95% CI]	*p*	HR [95% CI]	*p*
*R-ISS stage*				
I or II vs. III	0.37 [0.17–0.78]	0.01	0.13 [0.05–0.38]	<0.001
*MRD status (NGF)*				
Negative vs. positive	0.20 [0.09–0.45]	<0.001	0.21 [0.06–0.74]	0.02
*MRD status (NGS)*				
Negative vs. positive		>0.05		>0.05

Those variables with a significant impact in the univariate analysis were introduced in a multivariate Cox regression model to determine which ones were predictive for survival. Hazard ratios were calculated comparing first vs. second subgroup of each variable. Cytogenetics were included only as part of the R-ISS score.

*CI* confidence interval, *HR* hazard ratio, *MRD* minimal residual disease, *NGF* next-generation flow, *NGS* next-generation sequencing, *R-ISS* revised international staging system.

## Discussion

MRD evaluation has arisen as a very promising tool to predict the outcome of hematologic patients. On this behalf, the IMWG validated the use of this parameter to evaluate the deepest responses to treatment, measured either by flow or sequencing^16^. Moreover, the FDA has recently cleared the use of NGS to perform MRD analyses. However, to date only one strategy has been approved (Adaptive’s clonoSEQ), which makes access difficult for many centers, and raises the need to find other suitable strategies. Here, we have evaluated and compared two different strategies: NGF, developed by the EuroFlow group and recommended by the IMWG, and the NGS-based LymphoTrack® IGH panel, a commercial strategy designed by Invivoscribe Technologies.

MRD results are highly dependent on the quality and concentration of the samples, which is directly related to preanalytical conditions^9–11,20^. Since flow cytometry requires an immediate experimental procedure, this is usually taken as a disadvantage compared to NGS, whose samples can be frozen and stored after gDNA extraction for later analyses. On the other hand, NGS relies on the identification of clonotypic rearrangements at diagnosis, making that up to 10% of cases cannot be followed due to somatic hypermutation in primer-annealing regions, though this was not the case in this study, and once it is characterized, CDR3 regions represent a stable and reliable tool for MRD evaluation^29^. Other major flaws of NGS are the turnaround time, which is longer than for NGF (5–7 days compared to 24–48 h), and the need to run a high number of samples together. In contrast, interpretation of results is usually more difficult for NGF, requiring high expertise, while LymphoTrack’s solution is more user-friendly and semi-automated.

In our study, the use of ethanol precipitation resulted in suboptimal sample quantity, given that, in our experience, up to 40% of gDNA may be lost during its processing workflow, and the use of ethanol and sodium acetate usually turns to an increment of contaminating substances (salts and alcohol) that could interfere with PCR amplification. Accordingly, all the samples were optimal for NGF studies (criteria for inclusion), while 10% of the samples were suboptimal for NGS. If we had considered only optimal samples for both technologies, results would have favored NGS based on its slightly higher sensitivity using fewer cells; this represents a major advantage over NGF, that requires the acquisition of 10,000,000 events. However, all studies were done on an intention-to-study analysis, mimicking the intention-to-treat analysis that we usually see in clinical trials.

Despite being limited by the concentration method and the poor quality of certain samples, NGS was found to be fairly concordant with NGF in terms of MRD detection and quantification ability. Although we detected 15 discordant cases, only three of those patients relapsed (two positive cases by NGF, one positive case by NGS). In contrast, highly concordant results were observed in the MRD evaluation of the remaining 28 relapsing patients: 23 were double-positive and only five were negative by both methods, which could be explained by risk factors already present at diagnosis, extramedullary relapses^24^, or infiltration levels below the limit of detection.

Most discordant cases had MRD levels below 10^−5^, that may be explained either by a higher sensitivity for one method over the other, or in the case of NGS, if technical inaccuracies due to the use of the spike in for quantification were present (i.e. underestimation of the MRD level). The only six discordant cases (NGS = 3; NGF = 3) with MRD levels over 10^−5^ could be explained based on differences in the sampling procedure (different tubes from different bone marrow pulls), since sensitivity was set in the range of 10^−5^−10^−6^ for both techniques. Only three discordant cases relapsed, and accordingly no differences were seen with double-negative patients in terms of PFS, although an intermediate survival rate could be expected in a broader population with longer follow up.

According to the standard design of clinical trials, MRD is evaluated at several timepoints irrespective of the response. In our study, eight patients who had achieved VGPR as confirmed by immunofixation and electrophoresis had undetectable residual disease by next-generation techniques. This finding is concordant with a situation where tumor plasma cells had been eradicated in the bone marrow, while the monoclonal component was still present in the blood stream. Noteworthy, five of them achieved ≥CR after consolidation, supporting the notion that, in the clinical practice, MRD should be assessed whenever patients achieve CR^16,30^.

Cytogenetic status still maintained its potential to stratify patients, but only when applied to the MRD-positive subset, a feature that has already been described for patients treated with the combination of bortezomib, lenalidomide, and dexamethasone^31^. As far as this study is concerned, it seems that this treatment scheme could overcome the negative prognostic impact of t(4;14) and be able to provide MRD-negative responses. By contrast, 17p deletion still retained its association with poor survival rates, and was strongly correlated with MRD positivity.

Regarding the multivariate analysis, NGF retained a more independent informative capacity for survival than NGS because it was selected before in the multivariate analysis and the results of the two methods were closely associated. This result has to be interpreted with caution because the preanalytical conditions strongly affected NGS and could not be considered by the multivariate analysis. Despite that, NGS provided consistent and comparable results to NGF when only non-biased cases were evaluated.

As Perrot et al. have recently stated^14^, both NGF and NGS can reach a sensitivity of 10^−6^ when more cells are used and, in this context, the most important factor is not the technique but the ability to detect deeper levels of MRD, avoiding false-negative results due to lower detection limits. Direct comparison of MRD quantification by NGS and NGF in the same conditions (i.e. reaching the same sensitivity level and using the same marrow pull) should be performed to shed light into this issue, although an intrinsic false-negativity risk will always exist for both methodologies due to the patchy nature of the disease. As it was mentioned before, samples undergoing laboratory analysis are not the same, and a great variation can be detected from the first marrow pull (which is usually sent to the morphology laboratory) to the last one (which is distributed for molecular, cytogenetic, and immunophenotypic studies)^23,32^. This could be a feasible explanation for the 15 discordant cases in our cohort. Nonetheless, our study demonstrates the similarity between NGS and NGF, with analogous results to those reported in the GEM2012 overall analysis of MRD by NGF^30^ but using ~10 times less cells.

In summary, our results support the use of the LymphoTrack® strategy to detect and evaluate MRD in MM patients, with excellent applicability and comparable results to NGF. Altogether, these findings reinforce the use of MRD assessment as an endpoint in MM clinical trials and underline the need of standardization and quality assessment in future studies for all MRD approaches in MM.

## Supplementary information

Supplemental material
