# Supplementary material for: Comparison of next-generation sequencing (NGS) and next-generation flow (NGF) for minimal residual disease (MRD) assessment in multiple myeloma
Source: Blood Cancer J. 2020 Oct 30;10(10):108. doi: 10.1038/s41408-020-00377-0 (PMC7603393; doi:10.1038/s41408-020-00377-0)
Supplement: Supplementary file 1 — Supplemental material [file 41408_2020_377_MOESM1_ESM.docx]

**SUPPLEMENTARY MATERIALS**

|  | **FITC** | **PacB** | **PacO** | **PerCPCy5.5** | **PECy7** | **PE** | **APCH7** | **APC** |
| --- | --- | --- | --- | --- | --- | --- | --- | --- |
| **Tube 1** | CD38 | CD138 | CD27 | CD45 | CD19 | CD56 | CD81 | CD117 |
| **Tube 2** | CD38 | CD138 | CD27 | CD45 | CD19 | CD56 | CyIgλ | CyIgκ |

**Table S1. Eight-color antibody panel used for NGF-MRD evaluation.** APC: allophycocyanin; Cy: cytoplasmic; Cy5.5: cyanin5.5; Cy7: cyanin7; FITC: fluorescein isothiocyanate; Ig: immunoglobulin; MRD: minimal residual disease; PacB: pacific blue; PacO: pacific orange; PE: phycoerythrin; PerCP: peridinin–chlorophyll–protein.

|  | Frequency/Median [IQR] | |
| --- | --- | --- |
| Variable | Current study (N=106) | Rosinol et al. (N=458) |
| Sex  Men:  Women: | 57·5%  42·5% | 52·4%  47·6% |
| Age | 59 years [52-63] | 58 years [51-63] |
| IgH  IgG:  IgA:  Bence-Jones:  Non-Secretory: | 62·3%  27·4%  8·5%  1·9% | 60·0%  23·5%  15·2%  1·4% |
| IgL  Kappa:  Lambda: | 68·9%  28·2% | 64·0%  34·5% |
| Calcium | 9·4 g/dL [9·0-10·2] | 9·6 g/dL [9·0-10·0] |
| Creatinine | 0·9 mg/dL [0·7-1·1] | 0·9 mg/dL [0·7-1·1] |
| Albumin | 3·6 g/dL [3·2-4·0] | 3·8 g/dL [3·3-4·2] |
| β2 microglobulin | 3·8 mg/L [2·8-6·1] | 3·6 mg/dL [2·5-5·3] |
| Hemoglobin | 10·3 g/dL [9·1-12·0] | 10·9 g/dL [9·6-12·8] |
| Serum M protein | 3·7 g/dL [2·1-5·4] | 2·9 g/dL [1·3-4·6] |
| Urine M protein  Yes:  No: | 55%; 0·4 g/dL [0·2-1·1]  45% | 57%; 0·6 g/dL  43% |
| ISS  I:  II:  III:  Missing: | 34·0%  32·1%  31·1%  2·8% | 39·1%  36·2%  23·4%  1·3% |
| High LDH | 18·9% | 14·2% |
| t(4;14) | 11·4% | 14·1% |
| t(14;16) | 2·9% | 4·3% |
| 17p deletion | 9·5% | 9·8% |
| High-risk cytogenetics | 22·5% | 20·1% |
| 1q gains | 45·9% | 41% |
| 1p deletion | 7·1% | 7·7% |

**Table S2. Baseline characteristics of the patients.** Comparison of baseline characteristics between present (left) and global (right) series. H: heavy chain; Ig: immunoglobulin; IQR: interquartile range; ISS: international staging system; L: light chain; LDH: lactate dehydrogenase.

|  | **Diagnosis** | | | | **MRD** | **Relapse** | | |
| --- | --- | --- | --- | --- | --- | --- | --- | --- |
| **Patient** | **ISS** | **Elevated LDH** | **FISH** | **Extramedullary**  **Plasmacytomas** | **Depth of response** | **Extramedullary plasmacytomas** | **Monoclonal component** | **BM aPCs (%)** |
| **1** | I | No | SR | No | CR | No | No | 0·002 |
| **2** | II | No | HR | No | sCR | No | Yes | 17 |
| **3** | II | No | SR | No | sCR | Yes | No | 0 |
| **4** | II | No | SR | No | sCR | No | Yes | 6·9 |
| **5** | I | No | SR | Yes | CR | Yes | No | 0 |

**Table S3. Characteristics of patients with undetectable MRD who experienced disease progression.** BM aPCs: bone marrow abnormal plasma cells; CR: complete response; FISH: fluorescence in-situ hybridization; HR: high-risk cytogenetics [t(4;14), t(14;16) and/or TP53 deletion]; ISS: international staging system; LDH: lactate dehydrogenase; sCR: stringent complete response; SR: standard-risk cytogenetics.


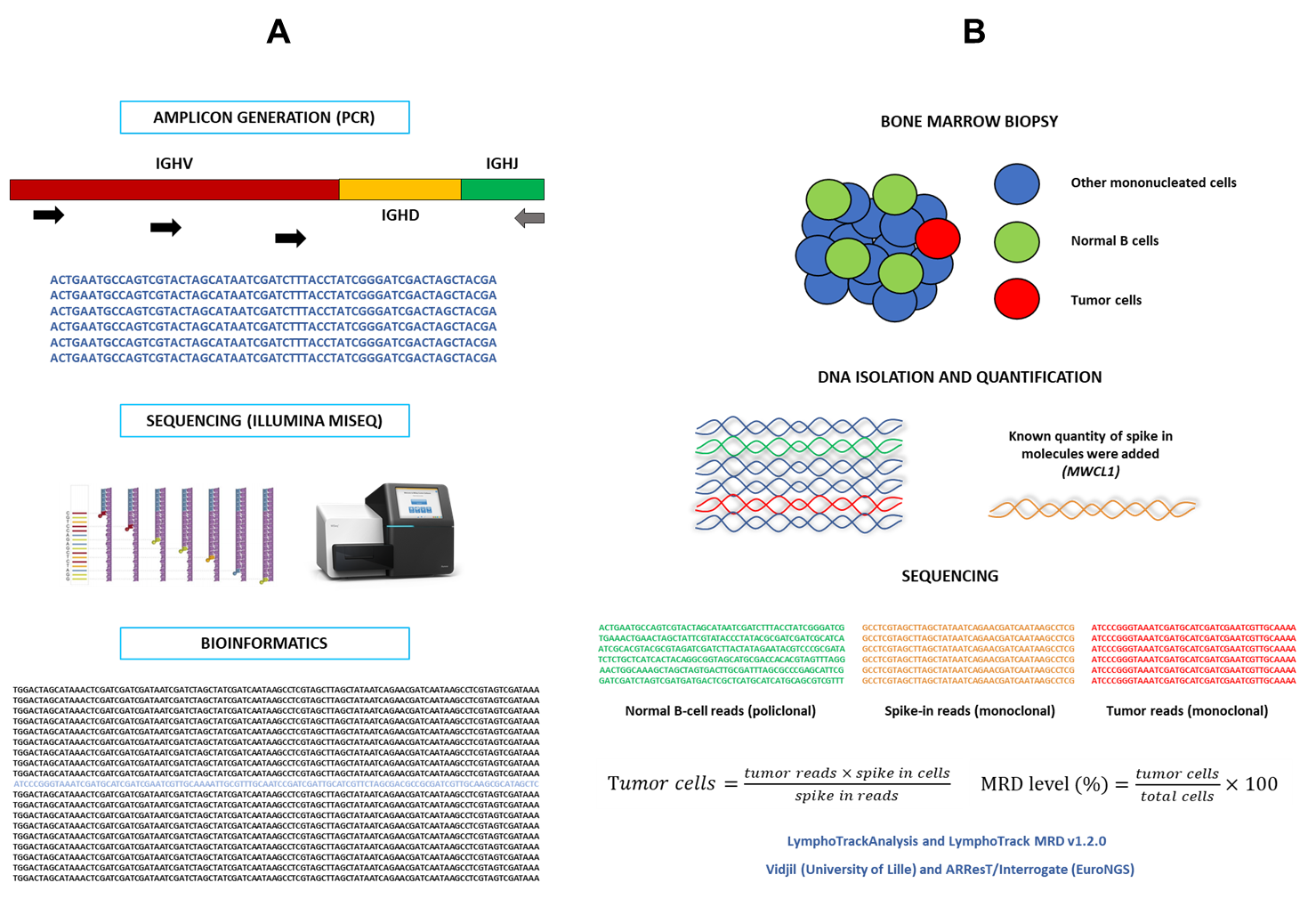


**Figure S1. NGS workflow.** A) Sequential steps for next-generation sequencing. Our NGS approach comprised three sequential steps: PCR amplification of VDJH rearrangements using the LymphoTrack® strategy, sequencing in the MiSeq platform, and bioinformatics analysis with FASTQ files. B) Bone marrow samples are comprised of normal B cells, tumor B cells and other mononucleated non-B cells. DNA from these cells is isolated and quantified with Qubit. When PCRs are assembled, a known quantity of spike in molecules is added in each reaction to quantify tumor cells. In our case, the MWCL1 cell line was used (kindly provided by Stephen Ansell, Mayo Clinic, Rochester, MN, USA). After sequencing, normal, spike-in and tumor reads can be distinguished and the number of tumor cells, as well as the MRD level, can be calculated.

**
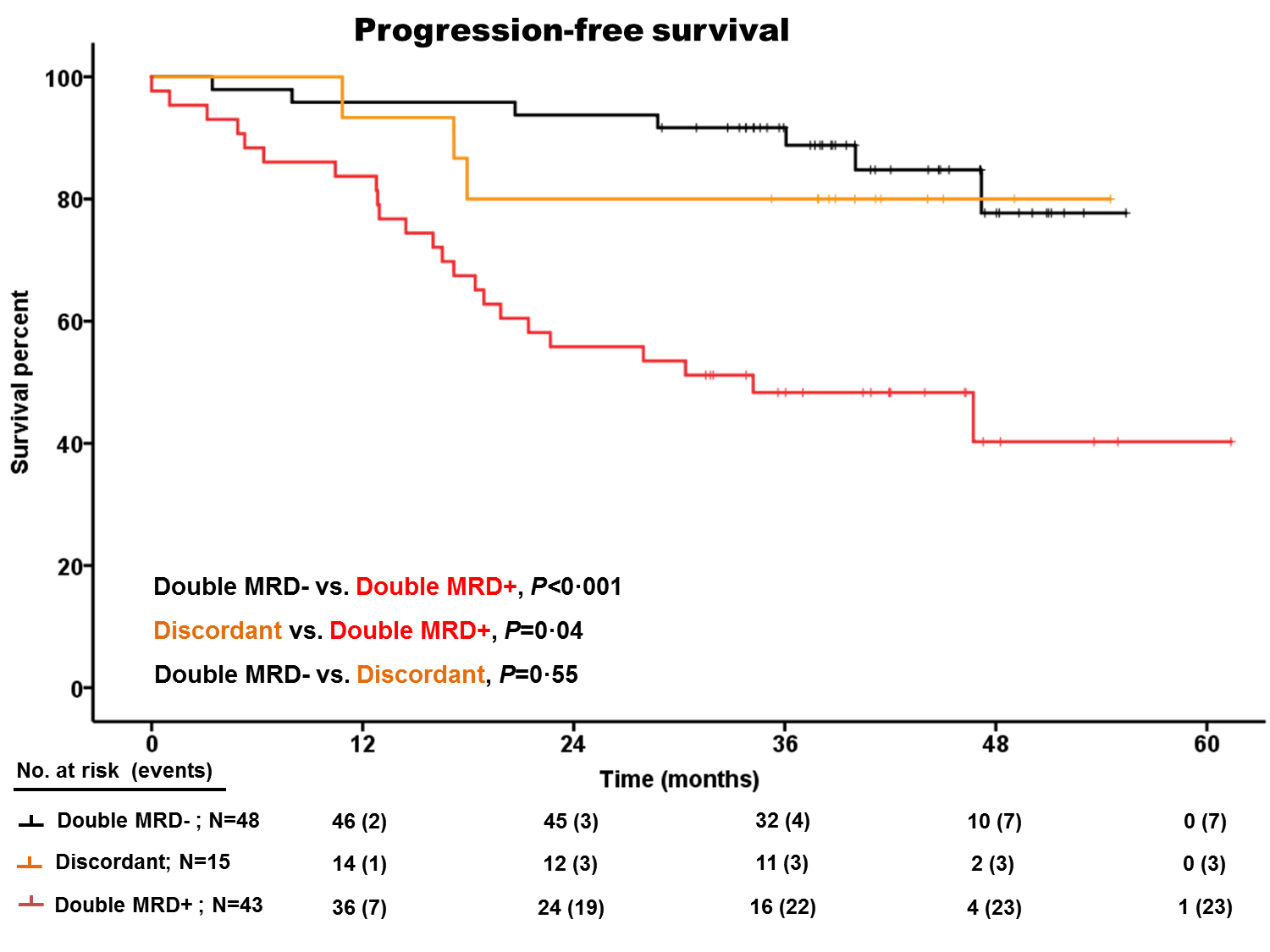
**

**Figure S2. Prognostic significance of combined MRD status.** PFS was calculated from the time of MRD assessment. Cases were stratified according to their MRD status as follows: double-negative (undetectable MRD by NGS and NGF, shown in black), discordant (positive MRD by one method, shown in yellow) and double-positive (detectable MRD by both NGS and NGF, shown in red). Patients at risk are shown at each point below the plot; events are shown between parentheses. There were no statistical differences between 36-month PFS rates of discordant and double-negative cases (91·7% vs. 80%, *p*=0·55), and both groups showed prolonged survival rates compared to double-positive patients (median PFS: 34·2 months).

**
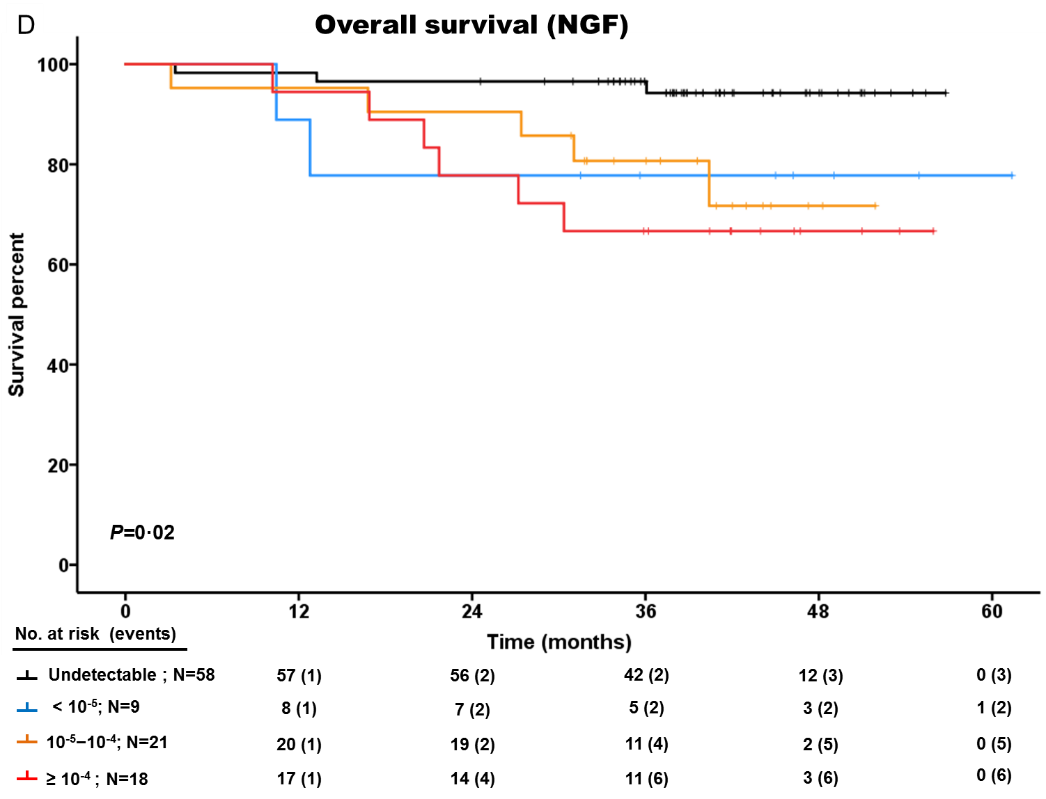

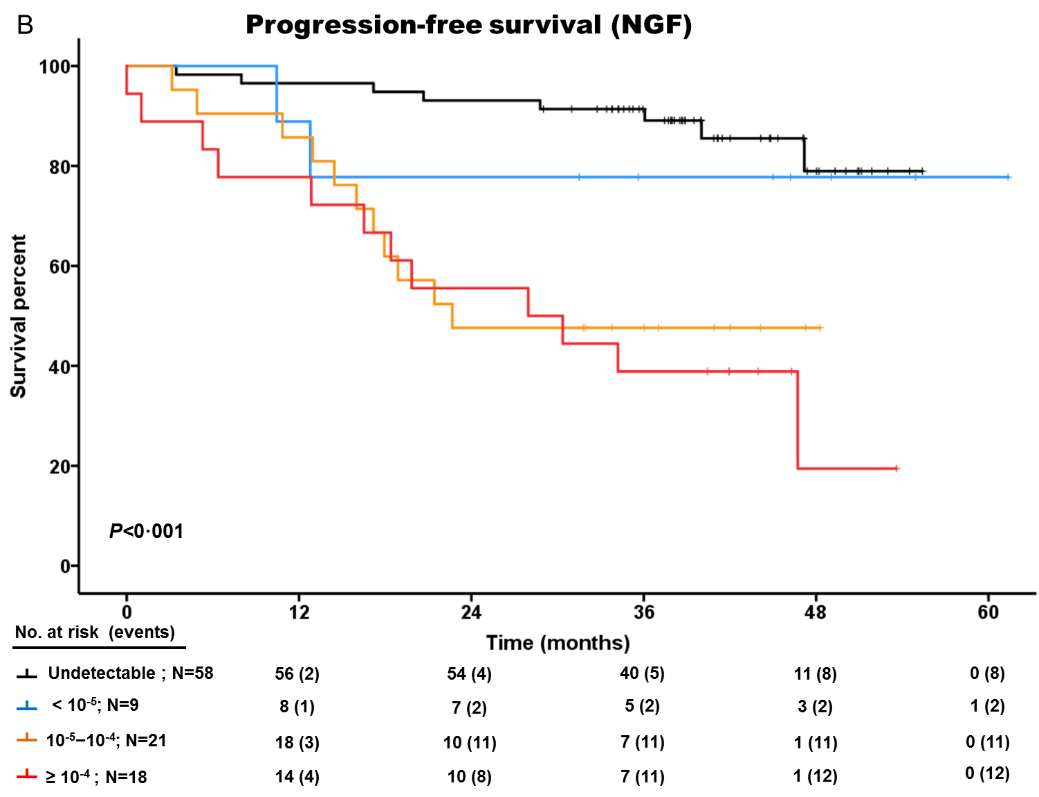

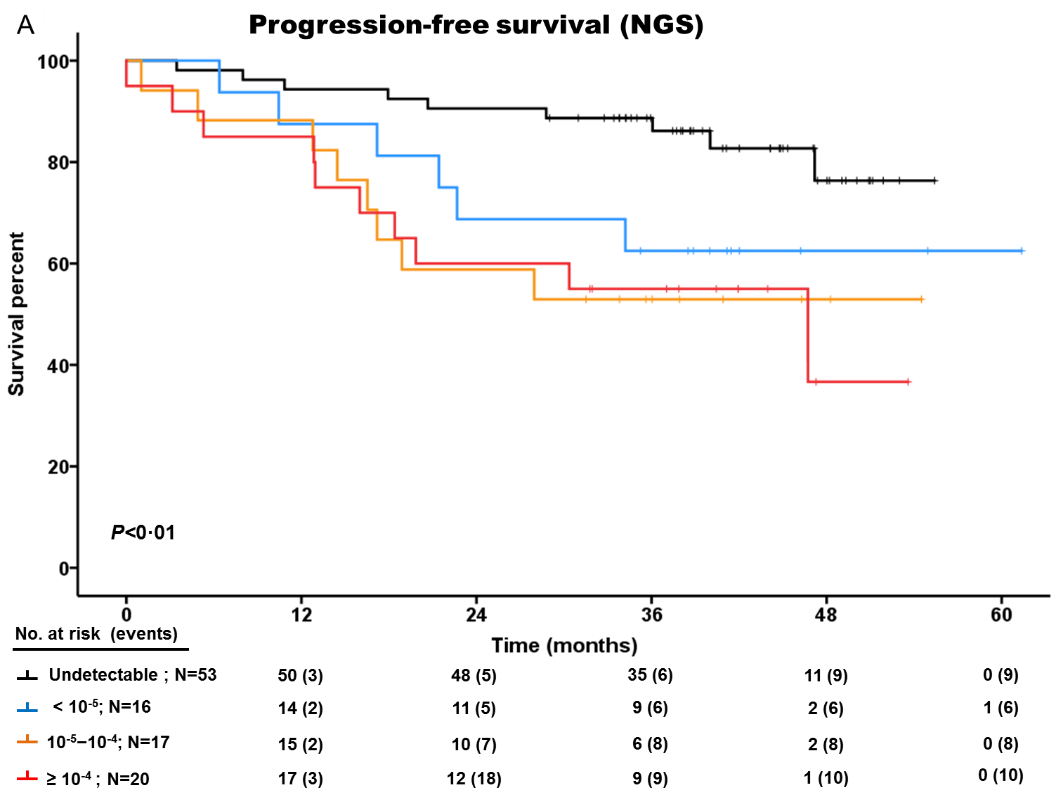

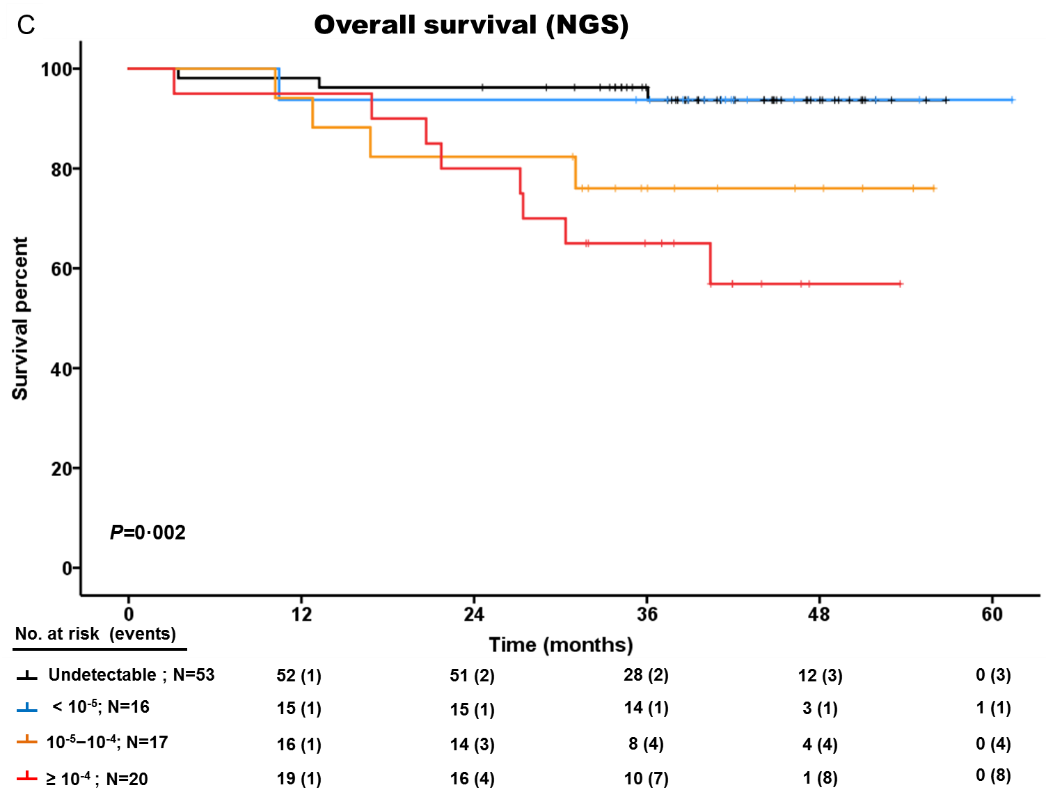
**

**Figure S3. MRD is relevant for survival at different levels.** Patients were stratified into four subsets based on their MRD levels: undetectable, <10^-5^, 10^-5^−10^-4^, ≥10^-4^. Progression-free (A and B) and overall survival (C and D) were plotted and compared for all groups. Patients from any group at risk are shown below each plot. The number of cumulated events is shown between parentheses. NGF: next-generation flow; NGS: next-generation sequencing.

**
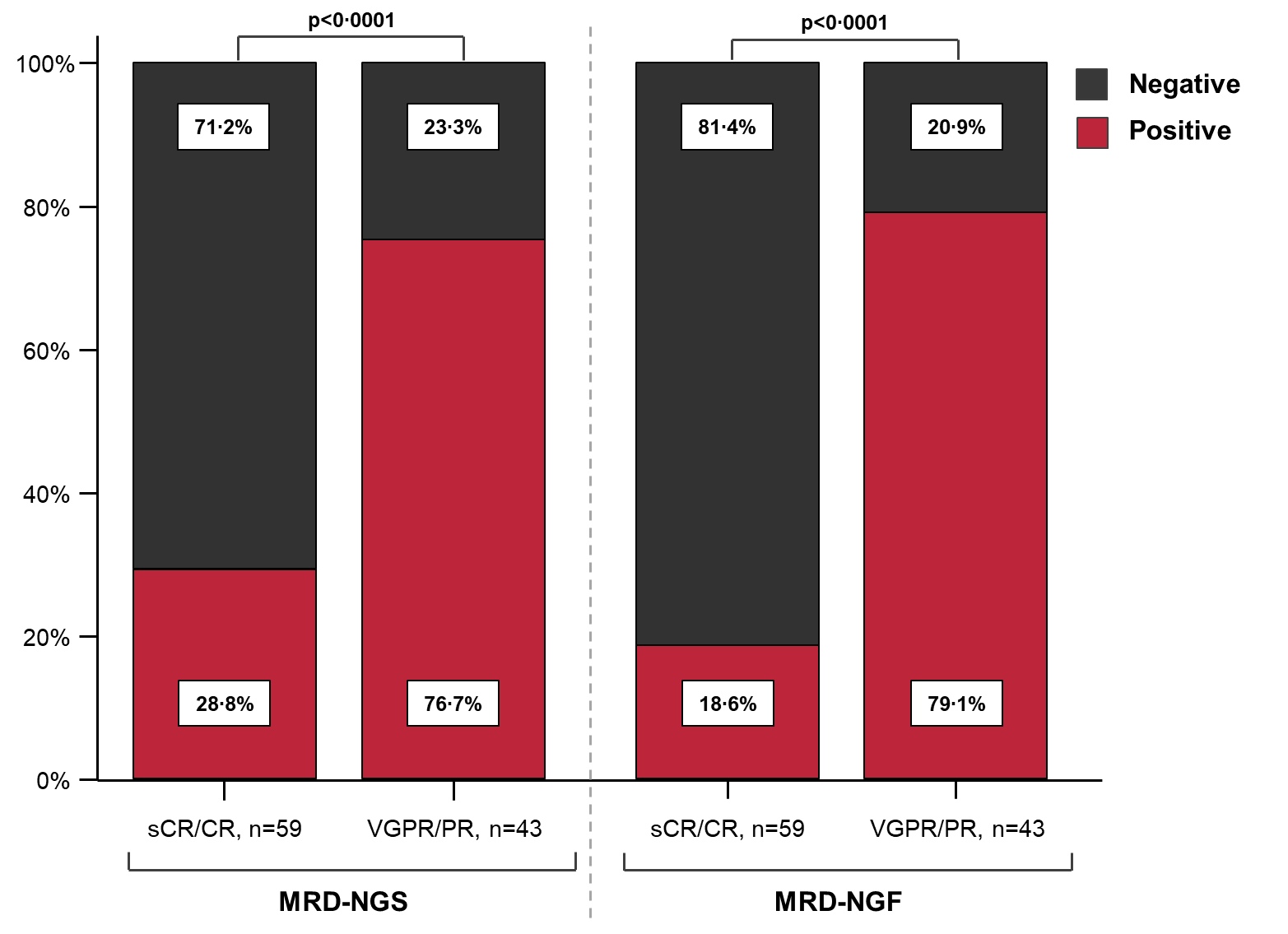
**

**Figure S4. Association between response to therapy and MRD status.** Responder patients (n=102) were grouped discriminating between those achieving complete response or better (n=59) and those achieving very good partial response or partial response (n=43), at the corresponding MRD evaluation time point that was assessed by next generation flow and next-generation sequencing. MRD-negative and MRD-positive cases are represented in black and red, respectively, with corresponding percentages in white squares. Statistically significant associations were observed between achieving MRD-negativity and optimal responses, either when flow or sequencing were used. No significant differences were observed between techniques (*p*>0.05). CR: complete response; MRD: minimal residual disease; NGF: next-generation flow; NGS: next-generation sequencing; PR: partial response; sCR: stringent complete response; VGPR: very good partial response.

**
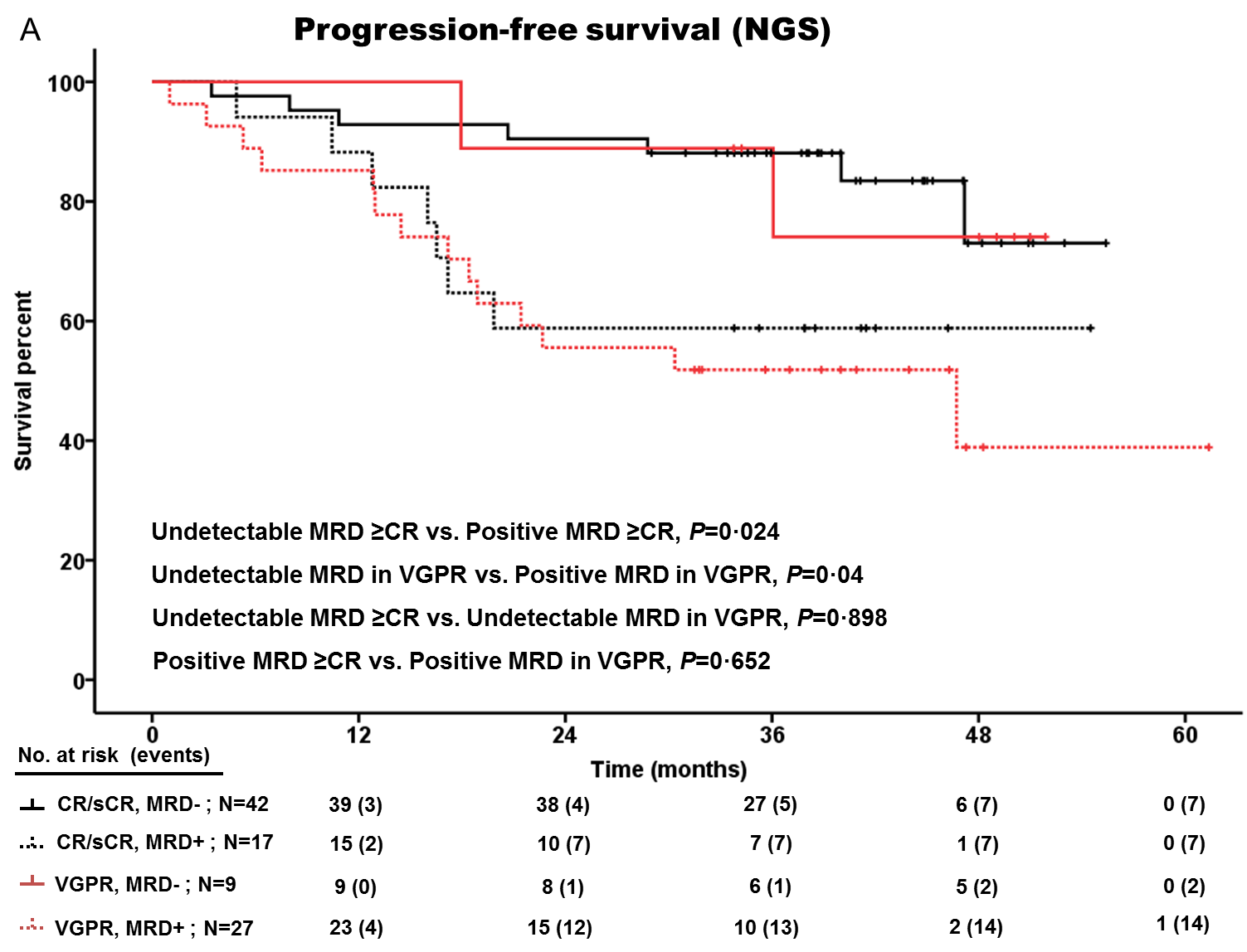

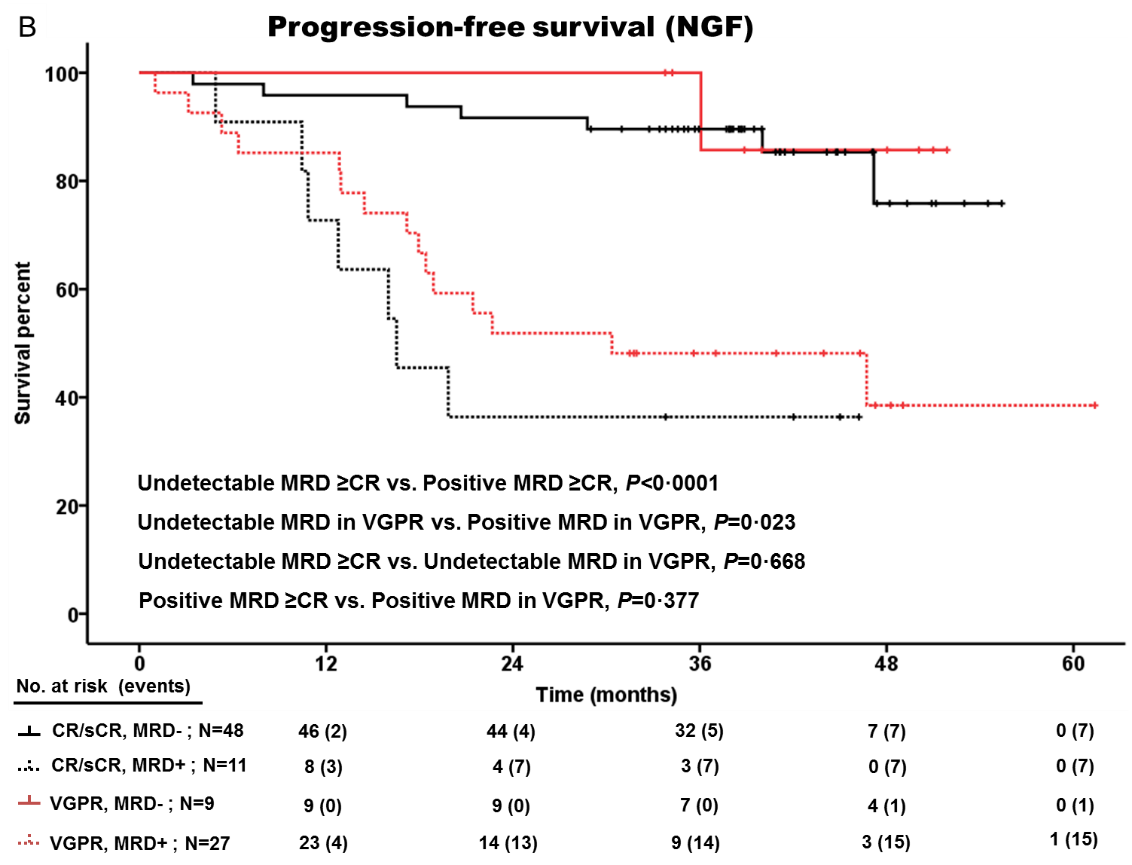
**

**Figure S5. Survival analysis comparing patients by the MRD status and conventional responses.** A) Progression-free survival for NGS-based results. B) Progression-free survival for NGF-based results. Patients achieving at least very good partial response at the corresponding follow up evaluation time point (n=95) were stratified according to the MRD status (positive and negative cases appear by dashed and solid lines, respectively), assessed by next-generation strategies, and the response (patients in ≥CR and VGPR are represented in black and red, respectively). Time was calculated from the time of MRD assessment, three months after transplantation. The number of patients at risk at any time point is shown below each plot (number of events appears between parentheses). CR: complete response; sCR: stringent complete response; MRD-: negative minimal residual disease; MRD+: positive minimal residual disease; VGPR: very good partial response.

**
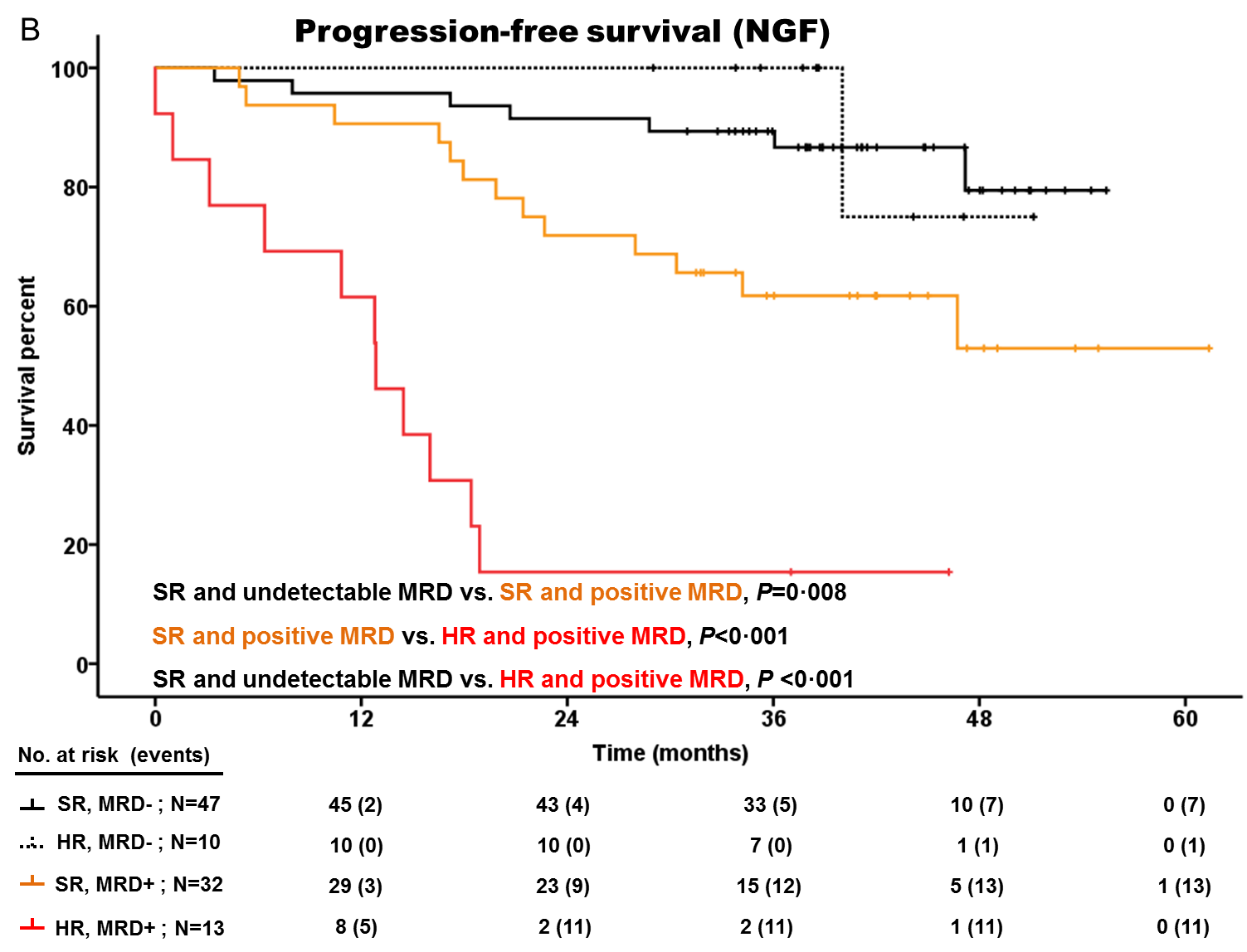

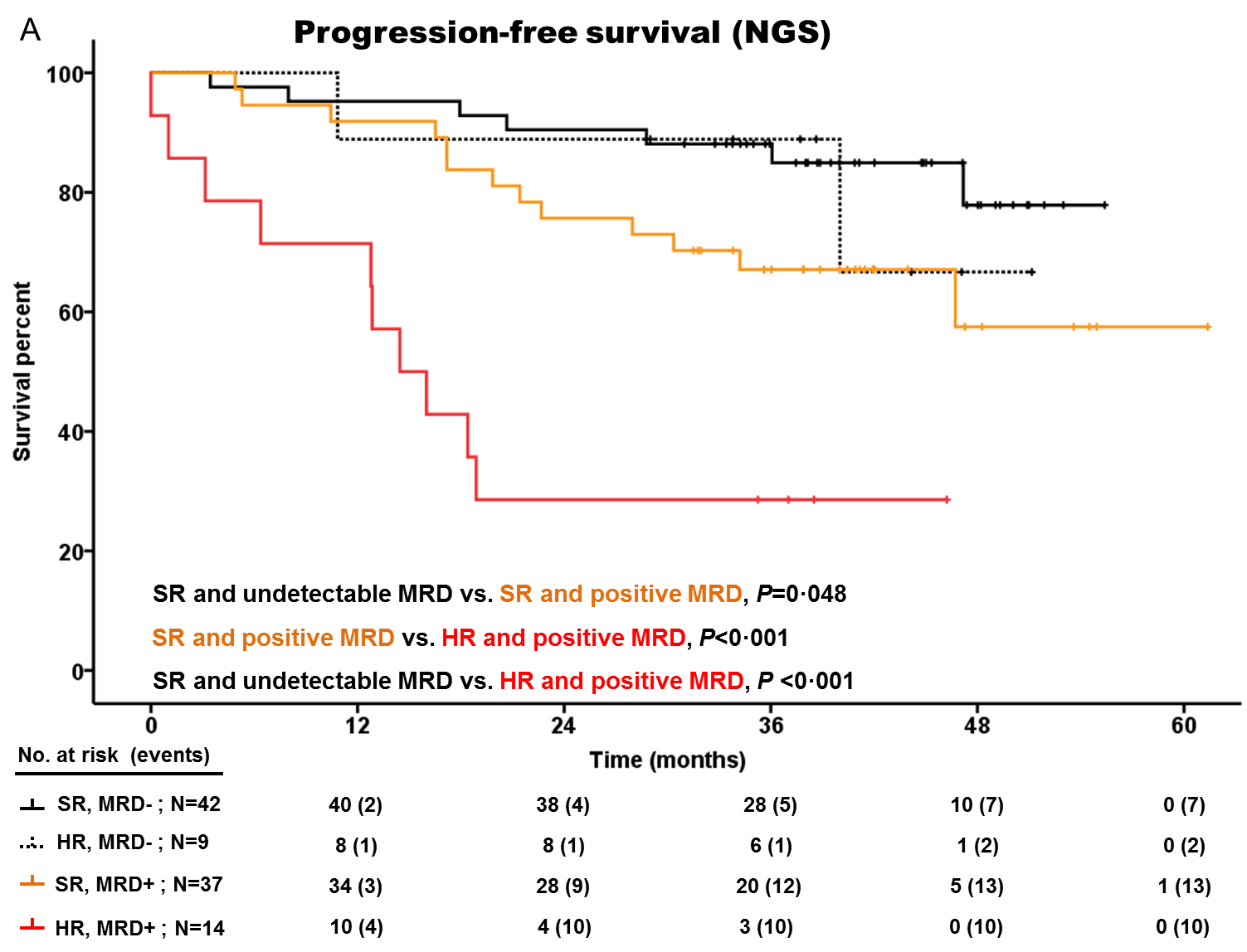
**

**Figure S6. Kaplan-Meier curves comparing progression-free survival according to the MRD status and the cytogenetic risk.** A) NGS-based results. B) NGF-based results***.*** Time was calculated from the time of MRD assessment, three months after transplantation. Patients with undetectable MRD are represented in solid black (standard risk) or dashed black (high risk), while patients with positive MRD are represented in orange (standard risk) or red (high risk). The number of patients at risk at any time point is shown below each plot (number of events appears between parentheses). There were no statistically significant differences in the risk of progression between MRD-negative patients, with 3-year PFS rates higher than 85% for all cases (p >0·1). MRD-positive patients without any high-risk cytogenetic alteration represented an intermediate risk group (three-year PFS 67·1% by NGS, 61·8% by NGF). Patients with combined MRD positivity and a high-risk cytogenetic profile showed poor performance, with a median survival of 14·5 months by NGS and 12·8 months by NGF. HR: high-risk cytogenetics; MRD-: negative minimal residual disease; MRD+: positive minimal residual disease; SR: standard-risk cytogenetics.


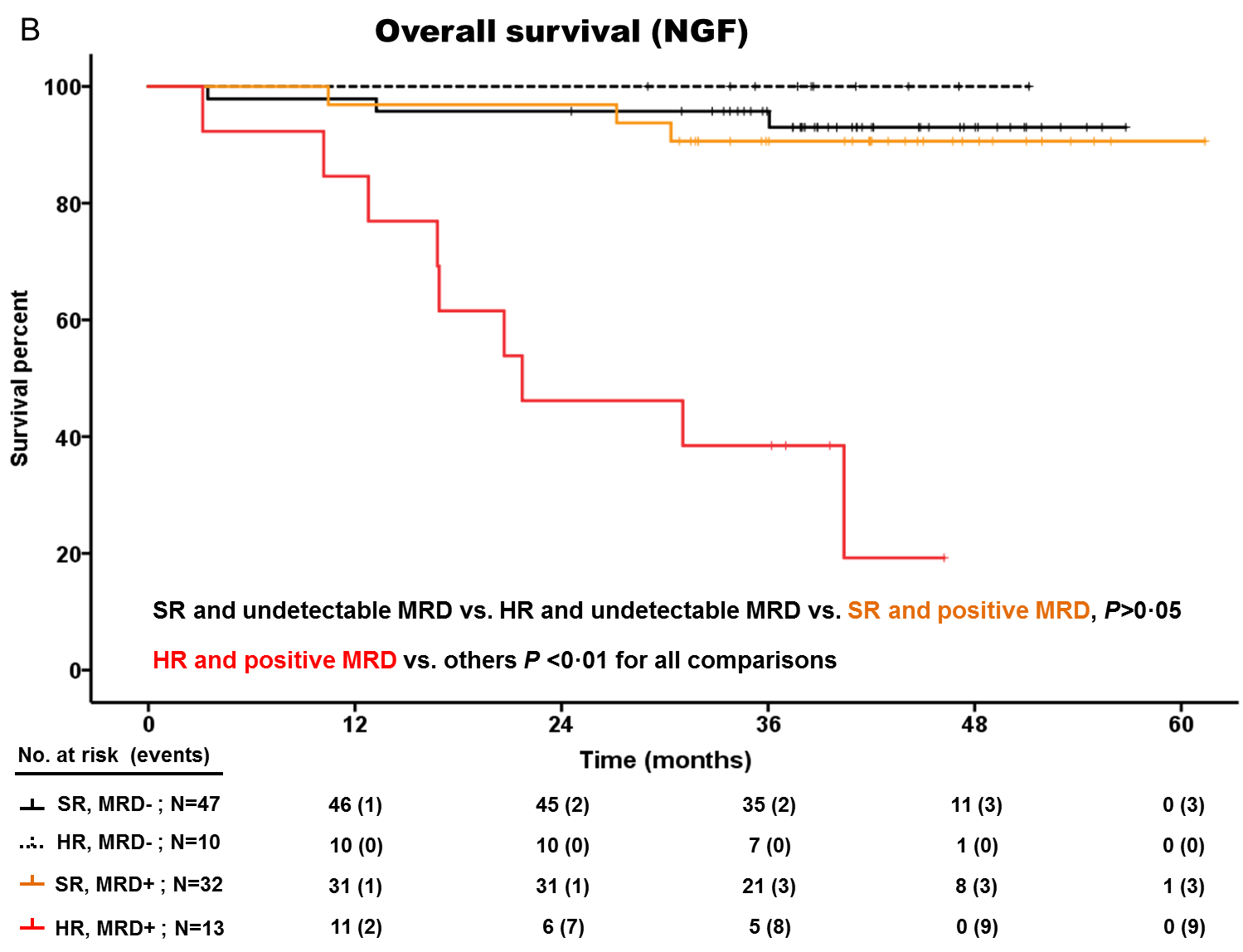

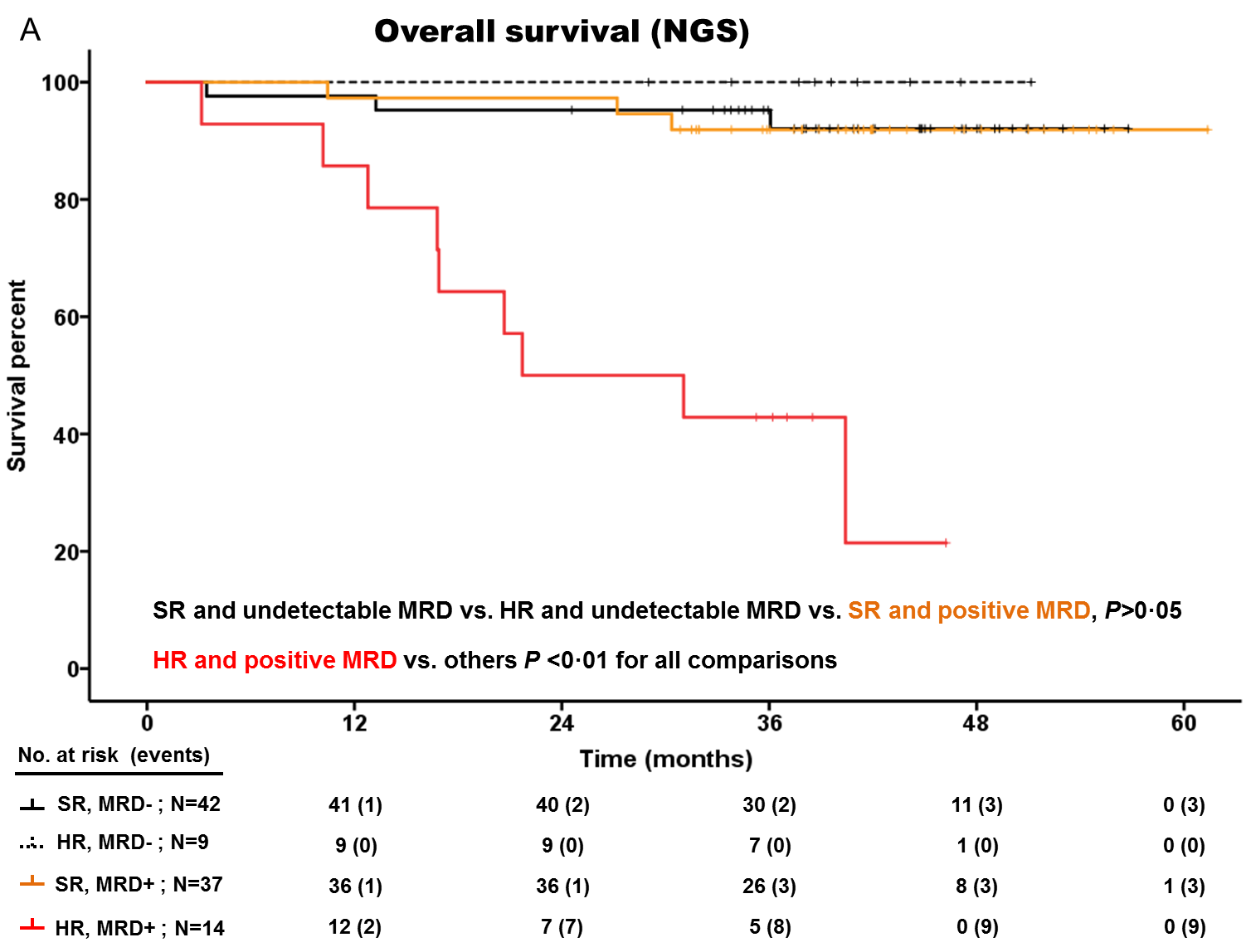


**Figure S7. Kaplan-Meier curves comparing overall survival according to the MRD status and the cytogenetic risk.** A) NGS-based results. B) NGF-based results***.*** Time was calculated from the time of MRD assessment, three months after transplantation. Patients with undetectable MRD are represented in solid black (standard risk) or dashed black (high risk), while patients with positive MRD are represented in orange (standard risk) or red (high risk). The number of patients at risk at any time point is shown below each plot (number of events appears in parenthesis). There were no statistically significant differences in the risk of progression between MRD-negative patients and MRD-positive patients with no high-risk cytogenetics, with 3-year OS rates higher than 90% for all cases (p >0·05). Patients with combined MRD positivity and a high-risk cytogenetic profile showed very poor survival rates, with a median survival of 21·7 months by NGS and NGF. HR: high-risk cytogenetics; MRD-: negative minimal residual disease; MRD+: positive minimal residual disease; SR: standard-risk cytogenetics.
